# HIV risk perception and testing behaviours among men having sex with men (MSM) reporting potential transmission risks in the previous 12 months from a large online sample of MSM living in Germany

**DOI:** 10.1186/s12889-016-3759-5

**Published:** 2016-10-22

**Authors:** Ulrich Marcus, Martyna Gassowski, Jochen Drewes

**Affiliations:** 1 Robert Koch-Institute, Department for Infectious Disease Epidemiology, Berlin, Germany; 2 Department of Public Health, Free University, Berlin, Germany

**Keywords:** HIV testing, Men having sex with men (MSM), Risk behaviour, Risk perception

## Abstract

**Background:**

HIV testing and serostatus awareness are essential to implement biomedical strategies (treatment as prevention; oral chemoprophylaxis), and for effective serostatus-based behaviours (HIV serosorting; strategic positioning). The analysis focuses on the associations between reported sexual risks, the perceived risk for HIV infection, and HIV testing behaviour in order to identify the most relevant barriers for HIV test uptake among MSM living in Germany.

**Methods:**

MSM were recruited to a nationwide anonymous online-survey in 2013 on MSM social networking/dating sites. Questions covered testing behaviours, reasons for testing decisions, and HIV risk perception (5-point scale). Additional questions addressed arguments in favour of home/ home collection testing (HT). Using descriptive statistics and logistic regression we compared men reporting recent HIV testing (RT; previous 12 month) with men never tested (NT) in a subsample not previously diagnosed with HIV and reporting ≥2 episodes of condomless anal intercourse (CLAI) with a non-steady partner of unknown HIV serostatus in the previous 12 months.

**Results:**

The subsample consisted of 775 RT (13 % of RT) and 396 NT (7 % of NT). The number of CLAI episodes in the last 12 months with non-steady partners of unknown HIV status did not differ significantly between the groups, but RT reported significantly higher numbers of partners (>5 AI partners: 65 vs. 44 %). While perceived risks regarding last AI were comparable between the groups, 49vs. 30 % NT were <30 years, lived more often in towns/villages <100,000 residents (60 vs. 39 %), were less out-particularly towards care providers-about being attracted to men (aOR 10.1; 6.9–14.8), more often identified as bisexual (aOR 3.5; 2.5–4.8), and reported lower testing intentions (aOR 0.08; 0.06–0.11).

Perceived risks (67 %) and routine testing (49 %) were the most common testing reasons for RT, while the strong belief not to be infected (59 %) and various worries (41 %) and fears of testing positive (35 %) were predominant reasons of NT. Greater anonymity (aOR 3.2; 2.4–4.4), less embarrassment, (aOR 2.8; 1.9–4.1), and avoiding discussions on sexual behaviour (aOR 1.6; 1.1–2.2) were emphasized in favour of HT by NT.

**Conclusions:**

Perceived partner knowledge and reasons reflecting perceived gay- and HIV-related stigma predicted testing decisions rather than risk perception. Access barriers for testing should be further lowered, e.g. by making affordable HT available, addressing structural barriers (stigma), and emphasizing beneficial aspects of serostatus awareness.

**Electronic supplementary material:**

The online version of this article (doi:10.1186/s12889-016-3759-5) contains supplementary material, which is available to authorized users.

## Background

HIV incidence among men having sex with men (MSM) has not been declining in larger European countries with published incidence estimates [1–3] despite favourable “treatment cascades” (a high proportion of people diagnosed with HIV are referred into care, initiate antiretroviral treatment, and achieve undetectable viral load) for MSM reported from Western Europe [1–7]. This is attributed to new infections occurring at a similar rate to that of diagnosis, resulting in a stable, not declining number of infected and untreated (because mostly undiagnosed) men [1]. Increasing condom use or reducing partner numbers would be necessary to reduce new infections-with both of these options being very difficult to achieve considering the diminished threat posed by a well treatable chronic HIV infection. Other options might be reducing the number of undiagnosed and untreated men with HIV infection by more frequent and better targeted HIV testing, or rolling out oral chemoprophylaxis for HIV (PrEP). However, initiating PrEP also requires prior confirmation of negative HIV status and frequent HIV re-testing. Identifying and removing or reducing barriers for HIV testing might thus be an essential requirement to lower HIV incidence among MSM in Europe.

According to findings of the European MSM Internet Survey (EMIS) 2010, a large Pan-European survey among MSM, and of the German follow-up survey SMA 2013 (“Schwule Männer und AIDS 2013”), more than 1/3 of the respondents never diagnosed with HIV tested for HIV within the previous 12 months, less than 1/3 tested longer ago, and approximately 1/3 have never been tested [8, 9]. Socio-demographic and some behavioural characteristics of non- and infrequent testers from several national EMIS 2010 samples and from SMA 2013 have been analysed and described [10–12]. Younger age (<25 years) and living in a settlement with less than 100,000 inhabitants was associated with lower testing coverage in all these analyses. In addition, in the most recent SMA 2013 survey infrequent and never testing was associated with lower reported partner numbers and less reported condomless anal intercourse (CLAI) in the previous 12 months compared to men who had been tested for HIV in the previous 12 months. However, despite this association, almost half of the survey participants reporting CLAI with a non-steady partner of unknown HIV serostatus in the previous 12 months reported either never having been tested for HIV or having been tested longer than 12 months ago. On the other hand, the majority of the survey participants reporting an HIV test in the previous 12 months did not report CLAI with a non-steady partner in this time span. Similar observations were reported from an analysis of HIV testing, risk perception, and behaviour in a representative sample of the British population, where the majority of MSM testing in the past year perceived themselves at no or very low risk for HIV. Large proportions of MSM with high risk perception had not been tested [13].

Several other studies among MSM report low partner numbers, no or few episodes of CLAI, and low perceived risks as reasons for not being tested [14, 15]. Since consistent condom use and restricting sexual encounters to few partners who have tested negative for HIV may be reasonable and effective risk management strategies to avoid HIV infection, we decided to compare not just factors associated with testing or not testing, but to compare such factors for men reporting similar levels of risk taking.

The research question for our analysis is, whether and how MSM reporting CLAI with non-steady partners who have been tested for HIV in the last 12 months differ from MSM who report the same risk behaviour and have never been tested for HIV. In the analysis we focus on number of partners, risk perception, outness, sexual identity, and reported testing preferences. A better understanding of risk perception and motivations associated with uptake or avoiding HIV testing in MSM at increased risk for HIV may help to devise improved and more effective strategies to promote HIV testing.

## Methods

Within the framework of the ongoing evaluation of the German national HIV/AIDS strategy, surveys on knowledge, attitudes and behaviours of MSM with regard to HIV (and other sexually transmitted infections) have been conducted in 2–4 year intervals since the late 1980ies [16, 17]. After the survey in 2010, which was integrated in EMIS 2010, a new survey was planned and conducted in 2013 (Schwule Männer und AIDS 2013-SMA 2013). Questions on HIV testing and risk perception were included in the 2013 SMA-survey, as well as questions on attitudes regarding home or home collection testing (both currently not available in Germany), and questions on the reason to decline a free testing voucher offered to all survey participants at the end of the questionnaire.

### Study procedures

Data were collected between November 2013 and January 2014 through a nationwide, anonymous online-survey targeting MSM (the SMA 2013 survey). Participants were recruited through private messages and banners on several social networking-and dating sites for gay and bisexual men. By clicking on a link or banner the participant was referred to the survey’s entry site, which contained information about the aims and contents of the survey, terms of participation and data privacy. By clicking on a button “I have read and understood the information above” the participant gave his informed consent and was referred to the online questionnaire. More details about study procedures including a CHERRIES checklist for the survey have been published as supplemental material in [9].

The online survey protocol was evaluated and approved by the ethical review board of the Charité University Clinic in Berlin (EA1/266/13). Suggestions by the data protection office of the federal state of Berlin to improve data protection for survey participants were implemented.

### Measures

We analysed responses to the following questionnaire items:

1. The socio-demographic variables age in years (categories 16–24, 25–34, 35–44, 45–54, 55 and above) and settlement size in number of inhabitants (categories <100,000, 100,000 – 1,000,000, >1,000,000); age was categorised because the relationship with testing is non-linear;
2. Self-defined sexual orientation (e.g. homo-, bi-, heterosexual) and outness about sexual orientation (“How many of the following people know that you feel attracted to men?”);
3. Perceived risk for HIV in the previous 12 months, and perceived risk at last anal intercourse (AI) with a non-steady partner; Perceived risks were queried on an 11-point scale from 0 (no risk) to 10 (very high risk). For analysis, the eleven-point scale was reduced to five risk categories (0–1 no/very low risk; 2–3 low risk; 4–5 moderate risk; 6–7 high risk; 8–10 very high risk), mainly because the sample size was too small for a meaningful statistical analysis of so many subgroups.
4. Intentions to test for HIV in the coming 12 months;
5. Reasons for non-acceptance of a voucher for free and anonymous testing at selected voluntary counselling and testing sites;
6. Reasons in favour of using a home/home collection test. One of the currently proposed options to increase HIV testing in groups at risk is home testing and/or home collection testing. Survey participants were asked several questions about their attitudes towards home/home collection testing for HIV, such as “Would you use a home/home collection test?”, if yes, “why would you use a home/home collection test?”
7. Risk management strategies used beyond condom use. Respondents could select from a list of items including a) partner well known; b) strategic positioning; c) withdraw before ejaculation; d) serosorting based on disclosed HIV test result; e) viral sorting based on reported viral load; f) partner appeared to be healthy; g) penis cleaning after anal intercourse.
8. Reasons for last testing and for never testing. The question on reasons for last testing had the following response options a) I experienced a transmission risk situation and wanted to check my status; b) I had symptoms suggestive of an acute HIV infection or suggestive of an AIDS illness; c) I was forced to test; d) I was recommended to test; e) I was testing to confirm HIV seroconcordance with my partner in order to stop using condoms; f) I get tested regularly; g) other reasons. If the respondent had never tested he was asked for his reasons not to test. Response options were a) I haven’t taken any risks so far; b) I have taken risks, but I don’t believe to have been infected; c) I don’t believe to be HIV-positive because my partner has tested HIV-negative; d) I am worried others might learn that I have sex with men; e) I’m worried others might think I am HIV-positive; f) I’m worried I will have to talk about the sex I have; g) I don’t want to be judged for the sex I have; h) I’m scared to have a test in case it is positive; i) I couldn’t stand the stress of waiting for the test result; j) I’m worried about confidentiality; k) There is no testing site I could go to near where I live; l) HIV test is too expensive for me; m) I’d rather not know my HIV status; n) I can still get tested later without losing treatment options; o) other reasons. For analysis, from the reasons for not testing reasons associated with worries, such as d), e), f), g), and j), were collated, reasons associated with accessibility and cost, such as k) and l), were collated, and reasons associated with testing-elicited fears, such as h) and i), were collated.

### Defining the population included in the analysis

We selected two subgroups for comparison: both subgroups did not report to be infected with HIV and reported two or more episodes of CLAI with a non-steady partner of unknown HIV serostatus in the previous 12 months. One subgroup had been tested for HIV in the previous 12 months (recent testers = RT); the other subgroup had never tested for HIV (never tested = NT).

### Statistical analysis

The two subgroups were compared regarding items 2) to 7) using multivariable logistic regression analysis, controlling for age and settlement size (1), and regarding item 8) using descriptive statistics.

## Results

### Sample description

The total study sample consisted of 16,734 MSM living in Germany, of these 15,297 had never received a positive HIV test result. Among these study participants, three distinct testing groups were identified: 5,340 (34.9 %) had never been tested for HIV, 5,885 (38.5 %) had been tested within the previous 12 months, and 4,072 (26.6 %) had been tested longer than 12 months ago. The two selected subgroups reporting CLAI with at least two non-steady partners in the previous 12 months consisted of 775 men who had been tested in the previous 12 months (13.2 % of all RT) and 396 men who had never been tested (7.4 % of all NT).

There were significant differences between the two subgroups regarding age and settlement size. In the NT group 62 % lived in a place with less than 100,000 inhabitants and almost 50 % were younger than 30 years; in the RT group 39 % lived in a place with less than 100,000 inhabitants and 26 % were younger than 30 years.

### Differences in sexual behaviour

Table 1 shows that despite reporting almost undistinguishable frequencies of CLAI with non-steady partners of unknown HIV serostatus, the two groups differed significantly in terms of absolute numbers of sex partners and AI partners in the last 12 months.

**Table 1:** Comparison of subgroups in terms of partner numbers for different partner characteristics in the last 12 months

Type of risk		HIV testing in the previous 12 months	Never tested for HIV	
		*n* = 775 n (%)	*n* = 396 n (%)	*p*-value
Sex with…	*2 to 5 partners*	164 (21.2)	149 (37.7)	0.000
	*6 to 10 partners*	184 (23.8)	111 (28.1)	
	*11 to 50 partners*	341 (44.1)	113 (28.6)	
	*>50 partners*	84 (10.9)	22 (5.6)	
Anal intercourse with…	*2 to 5 partners*	272 (35.1)	221 (55.8)	0.000
	*6 to 10 partners*	180 (23.2)	84 (21.2)	
	*11 to 50 partners*	279 (36.0)	76 (19.2)	
	*>50 partners*	44 (5.7)	15 (3.8)	
Condomless anal intercourse with…	*2 to 5 partners*	550 (71.0)	310 (78.3)	0.002
	*6 to 10 partners*	113 (14.6)	46 (11.6)	
	*11 to 50 partners*	100 (12.9)	28 (7.1)	
	*>50 partners*	12 (1.6)	12 (3.0)	
Condomless anal intercourse with a partner of unknown HIV status	*1–2 times*	226 (29.2)	109 (27.7)	0.823
	*3–10 times*	388 (50.2)	194 (49.4)	
	*At least once per month*	91 (11.8)	51 (13.0)	
	*(Almost) every week*	68 (8.8)	39 (9.9)	

### Differences in risk perceptions and outness

Perceived HIV risks, testing intentions, and outness about having sex with men are reported in Table 2. These variables were adjusted for age and settlement size.

**Table 2:** Risk perception, outness, sexual identity, intention to get tested, reasons to decline a test voucher, arguments in favour of home/home collection testing, and risk management beyond condom use in two MSM subgroups from Germany, 2013

		HIV test in the last 12 months (*n* = 775) n (%)	Never tested for HIV (*n* = 396) n (%)	OR	95 % CI	aOR^a^	95 % CI
Perceived risk at last AI	*No/very low risk (0–1)*	297 (40.97)	161 (43.40)	Ref.		Ref.	
	*Low risk (2–4)*	146 (20.14)	67 (18.06)	0.85	0.60–1.20	0.92	0.64–1.33
	*Medium risk (5)*	148 (20.41)	86 (23.18)	1.07	0.77–1.49	1.18	0.83–1.67
	*High risk (6–8)*	79 (10.90)	26 (7.01)	0.61	0.37–0.98	0.67	0.40–1.11
	*Very high risk (9–10)*	55 (7.59)	31 (8.36)	1.04	0.64–1.68	1.06	0.63–1.76
Perceived risk in the last 12 months	*No/very low risk (0–1)*	172 (24.09)	122 (33.89)	Ref.			
	*Low risk (2–4)*	237 (33.19)	109 (30.28)	0.65	0.47–0.90	0.69	0.48–0.97
	*Medium risk (5)*	183 (25.63)	79 (21.94)	0.61	0.43–0.86	0.59	0.40–0.85
	*High risk (6–8)*	83 (11.62)	26 (7.22)	0.44	0.27–0.73	0.42	0.25–0.71
	*Very high risk (9–10)*	39 (5.46)	24 (6.67)	0.87	0.50–1.52	0.80	0.44–1.44
Outness towards family doctor	*He/she knows*	383 (49.68)	43 (11.00)	Ref.		Ref.	
	*I don’t know or I think he/she knows*	154 (19.97)	61 (15.60)	3.53	2.29–5.44	2.98	1.90–4.66
	*He/she does not know*	174 (22.57)	243 (62.15)	12.44	8.59–18.02	10.07	6.86–14.78
	*I do not have a family doctor*	60 (7.78)	44 (11.25)	6.53	3.96–10.78	6.69	3.96–11.29
Outness towards parents	*At least one parent knows*	575 (84.43)	217 (66.77)	Ref.		Ref.	
	*Parents do not know*	106 (15.57)	108 (33.23)	2.70	1.98–3.68	2.63	1.88–3.67
Outness towards class mates/co-workers	*More than half know*	428 (56.69)	128 (33.95)	Ref.		Ref.	
	*Less than half know*	327 (43.31)	249 (66.05)	2.55	1.97–3.29	2.43	1.84–3.20
Self-definition	*Homosexual/gay/queer*	612 (79.27)	226 (57.22)	Ref.		Ref.	
	*Bisexual*	101 (13.08)	128 (32.41)	3.43	2.54–4.64	3.45	2.50–4.77
	*Heterosexual*	2 (0.26)	1 (0.25)	1.35	0.12–15.00	-	
	*Other/none*	57 (7.38)	40 (10.13)	1.90	1.23–2.93	1.64	1.03–2.61
Intention to get tested in the coming 12 months	*Yes*	653 (84.48)	121 (30.63)	Ref.		Ref.	
	*No*	120 (15.52)	274 (69.37)	12.32	9.23—16.46	12.68	9.28–17.33
Reasons to decline a free test voucher	*Time/distance*	93 (27.84)	61 (38.36)	1.61	1.08–2.40	1.23	0.79–1.90
	*Recent test*	160 (47.90)	2 (1.26)	0.01	0.00–0.06	0.02	0.00–0.06
	*Don’t perceive to be at risk*	46 (13.77)	35 (22.01)	1.77	1.09–2.88	1.53	0.91–2.58
	*No symptoms*	37 (11.08)	62 (38.99)	5.13	3.21–8.19	4.59	3.21–8.19
	*Currently don’t want to know*	12 (3.59)	38 (23.90)	8.43	4.26–16.66	7.75	3.81–15.78
	*Test not in a study context*	86 (25.75)	52 (32.70)	1.40	0.93–2.12	1.67	1.08–2.61
Arguments in favour of home/home collection testing	*More convenient and less time consuming*	516 (88.81)	215 (66.36)	0.25	0.18–0.35	0.24	0.17–0.35
	*Don’t want others to know that I’m testing for HIV*	133 (22.89)	169 (52.16)	3.67	2.74–4.92	3.21	2.35–4.38
	*Testing is embarrassing*	72 (12.39)	101 (31.17)	3.20	2.28–4.50	2.82	1.94–4.08
	*I don’t want instructions about my sex life*	134 (23.06)	104 (32.10)	1.58	1.17–2.13	1.56	1.12–2.16
	*I don’t need counselling*	167 (28.74)	54 (16.67)	0.50	0.35–0.70	0.58	0.40–0.84
Risk management beyond condom use Risk management beyond condom use	*Partner well known*	343 (45.61)	128 (34.04)	0.62	0.48–0.80	0.59	0.45–0.77
	*Undetectable viral load*	97 (12.90)	16 (4.26)	0.30	0.17–0.52	0.46	0.26–0.81
	*Only insertive*	316 (42.02)	94 (25.00)	0.46	0.35–0.61	0.54	0.40–0.72
	*Withdrawal before ejaculation*	271 (36.04)	103 (27.39	0.67	0.51–0.88	0.71	0.53–0.95
	*Partner appears healthy*	109 (14.49)	75 (19.95)	1.47	1.06–2.03	1.39	0.98–1.97
	*Partner tested HIV-negative*	293 (38.96)	106 (28.19)	0.62	0.47–0.80	0.54	0.40–0.72
	*Penis cleaning*	166 (22.07)	45 (11.97)	0.48	0.34–0.69	0.47	0.32–0.68

^a^Adjusted for age and city size

Analyzed sample consists of 1,171 participants with at least 2 cAI-partners in the last 12 months; 775 (66.18 %) were tested for HIV in the last 12 months, 396 (33.82 %) have never been tested for HIV. Outcome: HIV test: in the last 12 months (0); never (1)

The MSM who less often self-identified as gay/homosexual, were less out towards their family, their co-workers, and particularly their health care provider/family doctor, were more likely to have never been tested. In terms of risk perception, a larger proportion of NT men perceived their general risk for HIV in the last 12 months as low or very low. For the perceived risk of the last AI with a non-steady partner this difference between the two groups diminished; only the group reporting a high risk was significantly larger for the tested men. After adjustment for age and settlement size even this difference was no longer significant.

**Fig. 1:**
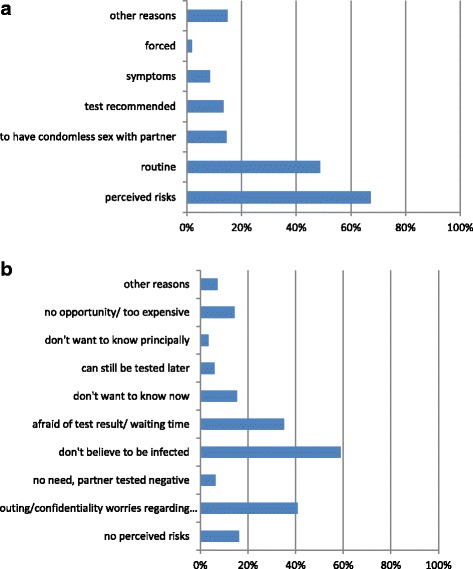
**a** Reasons for testing identified by recent testers. **b** Reasons for not testing identified by never testers

The notion of a similar-but not identical-risk perception is corroborated by the proportion of RT referring to perceived risks as a reason for testing (67 %) and the proportion of NT referring to the lack of perceived risks as reason for not testing (16 %) (see Fig. 1a, b), which is not completely, but close to complementary.

### Testing behaviours and testing preferences

Contrastingly, we found large differences in testing behaviour, intention to test, and reasons to test or not to test. Among the recent testers, 85 % intended to test again within the next 12 months, while 31 % of the never tested men intended to do so (see Table 2).

The main reason beyond perceived risks for RT to get tested was “routine testing”, stated by 49 % (see Fig. 1a). The main reasons for NT not to get tested were a strong belief not to be infected despite having taken some risks in the past (59 %), one or more from a variety of worries around risking to be outed as homosexual or somebody at risk for HIV, confidentiality breaches regarding the test result, or not wanting to discuss or disclose sexual behaviours with a counsellor (41 %), being afraid of the test result or the waiting time for the result (35 %), or not wanting to know a test result at the moment (21 %) (see Fig. 1b).

The proportion of RT and NT clicking on a link to receive a free test voucher offered at the end of the behavioural questionnaire was the same in each group (31 %), while the reasons for refusing the offered voucher were different (see Table 2). Apart from having been tested recently, the largest differences in reasons for not accepting the testing voucher were the lack of symptoms, and not wanting to know a test result, which were indicated as reasons more frequently by NT.

Regarding attitudes towards home/home collection testing, both subgroups most frequently mentioned convenience as a reason in favour of this testing option. Notably, reasons like “embarrassment” and “not wanting others to know about getting tested” were mentioned twice to thrice as often by NT (see Table 2).

### Differences in risk management

Reported risk management in both subgroups were broadly similar, with more NT men relying on their impression of the partner (appears healthy), and more RT men using serostatus knowledge-dependent risk management strategies (HIV serosorting, strategic positioning, viral sorting) (see Table 2). Notably, a considerable proportion of NT men reported risk management strategies requiring serostatus knowledge for being effective, such as not using condoms with partners who disclosed to have tested negative for HIV, a behaviour termed HIV serosorting for people who have themselves tested negative for HIV, and strategic positioning (being the insertive partner during anal intercourse).

## Discussion

When comparing subgroups of recently and never tested MSM who report similar levels of sexual risk taking, risk perception between these two subgroups appears quite similar-more so for the last AI than for sexual risks taken within the previous 12 months. The slightly lower risk perception in the never tested group regarding risks taken within the last 12 months may be explained by a lower number of sex partners in the untested group. This suggests that the number of different partners is more important for risk perception and testing decisions than the frequency of unprotected anal sex acts with these partners. Knowing the partner well was the most common reason mentioned in both groups for not using condoms with a non-steady partner.

Further arguments for very similar risk perceptions between the two groups are the almost equal proportions in both groups willing to accept free test vouchers. Voucher acceptance represents rather a theoretical intention than a concrete action and thus appears to be closer linked to risk perception than actual testing.

However, while perceived risks and-if risk perception is low-an acquired habit of routine testing resulted in testing in the RT group, worries of being outed as gay, recognized as someone at risk for HIV, of needing to disclose personal secrets to a counsellor or health care provider, and fears of receiving a positive test result dominated the decision process and prevented men in the NT group from testing, despite of similar risk perceptions. A Scottish study [18] also concluded that compared with those tested within the previous year, those never tested had greater fear of a positive-HIV test result and a weaker norm for HIV testing. In the Scottish study-as in ours-condomless anal intercourse (CLAI) did not discriminate among the HIV testing groups. The authors highlighted the need to promote HIV testing in Scotland among those with high fear of testing, and those whose sexual behaviour puts them at risk. They recommended that interventions to increase HIV testing should promote positive norms and challenge the fear of a positive result.

Self-identified gay men who are out about their sexual orientation towards their social environment seem to be responsive to recommendations for routine testing of MSM, even if they perceive their risk as low. Contrastingly, men who do not self-identify as gay and are not out towards their close contacts are far less likely to have ever had a test. These results are comparable to reasons for not testing reported by Margolis et al. [19]. Predictors of never being tested identified in his study included younger age (18–24), bisexual or heterosexual orientation, living outside of large metropolitan areas and not having a healthcare provider.

In the attitudes towards home/home collection testing, the same issues come up again: among the reasons in favour of home/home collection testing the never tested men agree with the reasons “increased anonymity” and “less embarrassing” twice to three times as often as men recently tested (50 vs. 20 % for anonymity, 30 vs. 10 % for less embarrassing).

### Limitations

While we tried to construct the comparison groups based on comparable numerical sexual risk criteria, we did not have sufficient data to characterise the sexual partners and to differentiate e.g. between anonymous casual partners and well-known sex friends. Consequently we are unable to rule out that the two groups might differ substantially regarding the types of non-steady partners they have CLAI with. Since all data were self-reported, the usual limitations of self-reported data such as recollection bias and social desirability bias need to be recognized.

## Conclusions

Based on our analysis we conclude that differences in HIV testing behaviours among MSM are not primarily due to differences in risk perception, but rather due to a combination of differences in number of partners-not number of CLAI episodes-and perceived individual benefits and costs of HIV testing. Consequently, one approach to reduce the proportion of never and infrequently tested MSM would be the lowering of access barriers for testing, e.g. by making home collection or home tests available.

However, this may not turn out to be a “magic bullet”, as the psychosocial barriers for testing may end up being exchanged for financial barriers due to the relatively high prices for home tests, and since the social and psychological consequences of testing positive may still be more frightening to some men living “in the closet”. In addition it will be necessary to emphasize health benefits of early diagnosis and to re-assure people at risk regarding the anonymity respectively confidentiality of testing and diagnosis.

It seems to be possible to override a lack of perceived risk by establishing community norms for routine testing. However, this approach becomes more challenging when dealing with MSM who do not self-identify as part of a gay community.

To verify our conclusions, it may be useful to monitor risk perception, community involvement, perceived individual benefits and costs, and previous HIV testing history of participants and users of respective pilot studies or programme roll-outs.

One approach to deal with inadequate risk perceptions may be to emphasize routine testing independent of sexual self-identification, except for those who definitely did not have risks. Not undergoing risks would need to be clearly defined, e.g. as not having had anal or vaginal intercourse or using condoms consistently with every partner. However, this may lead to an unintended increase of unnecessary concerns in low-risk heterosexual populations. Also it may be very difficult in countries with no generalized HIV epidemic to effectively challenge the notion that steady or well-known partners pose much lower risks for HIV than non-steady partners, except for some very specific sub-populations among gay and bisexual men.

## Additional file

Additional file 1: A subset of the survey dataset including the variables used for the analysis and the data analysis syntax (stata do-file) are provided as Additional File 1 (Zip-archive, including a CSV-data file and the do-file in txt-format). (7Z 111 kb)

## Abbreviations

AI: anal intercourse
AIDS: Acquired Immuno Deficiency Syndrome
aOR: Adjusted Odds Ratio
CLAI: Condomless anal intercourse
EMIS: European MSM Internet Survey
HIV: Human Immunodeficiency Virus
HT: Home/home collection testing
MSM: Men having sex with men
NT: Never tested for HIV
PrEP: (HIV) Pre-exposure prophylaxis
RT: Recent HIV testing
SMA: “Schwule Männer und AIDS”-Survey
